# A new sponge from the Marjum Formation of Utah documents the Cambrian origin of the hexactinellid body plan

**DOI:** 10.1098/rsos.231845

**Published:** 2024-09-18

**Authors:** Lucas Del Mouro, Rudy Lerosey-Aubril, Joseph Botting, Robert Coleman, Robert R. Gaines, Jacob Skabelund, James C. Weaver, Javier Ortega-Hernández

**Affiliations:** ^1^ Department of Organismic and Evolutionary Biology and Museum of Comparative Zoology, Harvard University, Cambridge, MA 02138, USA; ^2^ Institute of Geosciences, University of São Paulo, São Paulo 05508-080, Brazil; ^3^ Amgueddfa Cymru, National Museum Wales, Cardiff CF10 3NP, UK; ^4^ Nanjing Institute of Geology and Palaeontology, Nanjing 210008, People’s Republic of China; ^5^ Unaffiliated, Round Lake Beach, IL 60073, USA; ^6^ Geology Department, Pomona College, Claremont, CA 91711, USA; ^7^ Unaffiliated, Wellsville, UT 84339, USA; ^8^ Wyss Institute for Biologically Inspired Engineering, Harvard University, Boston, MA 02218, USA

**Keywords:** Porifera, Hexactinellida, body plan, Cambrian, Marjum Formation, Konservat-Lagerstätte

## Abstract

Modern poriferans are classified into four classes—Calcarea, Demospongiae, Hexactinellida and Homoscleromorpha—the recognition of which in fossil specimens almost exclusively relies on spicule morphology and arrangement. Early fossil representatives of the phylum Porifera are morphologically diverse, and many of them problematically display characteristics that are incompatible with the classification scheme developed for modern taxa. Critically, hexactine spicules—a diagnostic feature of hexactinellids among modern taxa—are found in various Cambrian and Ordovician taxa that cannot be accommodated within the hexactinellid body plan. Here we describe a new poriferan from the Drumian Marjum Formation of Utah, *Polygoniella turrelli* gen. et sp. nov., which exhibits a unique combination of complex anatomical features for a Cambrian form, including a syconoid-like organization, a thick body wall, and a multi-layered hexactin-based skeleton. The hexactinellid-like body wall architecture of this new species supports a Cambrian origin of the hexactinellid body plan and provides valuable insights into character evolution in early glass sponges.

## 1. Introduction

Poriferans, colloquially known as sponges, are substantial contributors to the biodiversity (*ca* 8300 species) [1] and biomass (e.g. [2]) of modern marine ecosystems, and their significance in marine communities is predicted to increase in response to climate change [3–5]. Despite the anatomical simplicity of sponges, these predominantly filter-feeding benthic organisms represent formidable ecological engineers that directly or indirectly impact key aspects of the functioning of the oceanic ecosystem, such as bioerosion, geochemical cycles, nutrient transfer, primary production, and turbidity [6–10]. The ecological significance of sponges in marine environments has its origins in deep time, as sponges have played instrumental roles during pivotal periods of the biosphere’s history. For example, sponges likely facilitated the recovery of marine life in the aftermath of mass extinctions [11–14], and they have been directly implicated in the emergence of the modern Phanerozoic biosphere [15,16] via their role in promoting an oxygenated benthic environment.

Sponges have a rich fossil record in the Cambrian and include both spiculate taxa and the extinct hypercalcified group Archaeocyatha, which are structurally similar to the early demosponges of the family Vauxiidae [17]. Spiculate sponges are particularly well represented in sites of exceptional preservation around the world, particularly in Burgess Shale-type deposits where they typically represent one of most diverse components of the fossil biotas (e.g. [18–22]). Early poriferans exhibit impressive morphological disparity, and regularly feature combinations of characters that cannot be accommodated into the classification scheme developed for stratigraphically younger representatives (e.g. [23–25]). Increasing awareness of this fact has led to the reinterpretation of lower Palaeozoic forms as phylogenetically distant from the crown lineages of modern classes (contra [26]). Most early Palaeozoic sponges are now considered by some authors as stem-group members of modern classes, the superclade Silicea (i.e. Demospongiae and Hexactinellida), or even the whole phylum Porifera (e.g. [27–29]). Although several crown-group middle Cambrian demosponges (i.e. hazeliids and vauxiids) have been identified [30,31], the oldest definite evidence of crown-group hexactinellids dates back only to the basal Ordovician (isolated microscleres) [32] and latest Ordovician (articulated sponges) [33]. The study of lower Palaeozoic sponge fossils suggests that some skeletal characteristics regarded as key for the classification of living sponges are of limited use, if not misleading, for reconstructing poriferan phylogenetic relationships [27]. For example, triaxial spicules with six rays—or hexactins—and their derivatives with fewer rays at 90° angles are diagnostic of modern hexactinellids, but such spicules can be found in a great variety of early Palaeozoic taxa displaying other features incompatible with an assignment to crown-group Hexactinellida (e.g [28–30,34–37]). Although many of the documented examples are Ordovician in age, they generally represent taxa belonging to groups that dominate Cambrian soft-bottom communities.

Such ambiguous status is the case for ‘reticulosans’, a group of lower Palaeozoic sponges characterized by a thin-walled body and a distinctly reticulate (i.e. net-like) biomineralized skeleton made of hexactins. Created as an extinct hexactinellid order [38], Reticulosa is now regarded by many authors as an artificial entity defined using a set of plesiomorphic characters, and potentially including stem-group and questionably crown-group hexactinellids, stem-group demosponges, stem-group siliceans and possibly even stem-group poriferans [27]. Hexactins have also been documented in the Cambrian heteractinid *Eiffelia* [34]. This is a member of a wider group that includes universally accepted stem-group calcareans such as the Astraeospongiidae [39], and even though it is possible that *Eiffelia* represents a slightly deeper position near the base of the poriferan crown group, this observation definitively demonstrates that the presence of hexactins alone does not permit a confident assignment of early sponges to total-group Hexactinellida, let alone its crown lineage.

In this contribution, we describe *Polygoniella turrelli* gen. et sp. nov., a new thick-walled sponge from the middle Cambrian (Drumian) Marjum Konservat-Lagerstätte of Utah, which combines a hexactin-based biomineralized skeleton and a hexactinellid-like body plan and aquiferous system. The discovery of a Cambrian sponge with a body wall architecture comparable to modern hexactinellids suggests a Cambrian origin of the hexactinellid body plan, and illuminates the character polarity implicated in the early evolutionary history of this important poriferan group.

## 2. Material and methods

### 2.1. Material

The studied material is predominantly composed of fossils (*ca* 190 specimens) collected by a team led by R.L.-A. and J.O.-H. at the Gray Marjum site in the House Range of western Utah, USA. This 50 m^2^ quarry on US federal lands was opened in 2022 (Bureau of Land Management (BLM) permit to R.L.-A. and J.O.-H.) and is located near the mouth of the Wheeler Amphitheatre, *ca* 2 km northwest of the Antelope Spring Reservoir (electronic supplementary material, figure S1). The Gray Marjum section exposes a 6.5 m-thick succession of light grey thin-bedded calcareous mudstone *ca* 125 m above the base of the formation. This material was complemented with 10 specimens deposited by the BLM at the Natural History Museum of Utah, Salt Lake City. The precise geographic origin and stratigraphic position within the formation of these additional specimens were not recorded, but their preservation (dark grey carbonaceous compressions) and the lithological characteristics of the matrix surrounding them (light grey mudstone) are strongly reminiscent of material from the geographically restricted Gray Marjum area. A complete list of the studied material is provided as electronic supplementary material, data S1.

The Marjum Formation consists of up to 430 m of thin-bedded (Marjum Pass area) to thick-bedded (Swasey Mountain) limestone interbedded with mudstone and marl, and is geographically limited to the House Range of Utah (electronic supplementary material, figure S1, and Foster & Gaines [40]). With the underlying Wheeler Formation and overlying Weeks Formation, it belongs to a continuous succession of deposits that filled the House Range Embayment, a fault-controlled basin that formed locally within the offshore margin of a carbonate platform in the late Wuliuan and persisted to the Guzhangian [40–42]. In this deep-water quiet marine environment, oxygen-poor conditions repeatedly developed, helping to facilitate the preservation of organic remains [43], as documented in the House Range by no less than three Konservat-Lagerstätten (one in each formation) [20,44]. The exceptionally preserved Marjum Biota is upper Drumian in age (*Ptychagnostus punctuosus* agnostoid biozone) and includes more than a hundred species, which inhabited the subequatorial northern margin of the palaeocontinent Laurentia [44–50].

### 2.2. Institutional abbreviations

The studied fossils are housed in the collections of the Museum of Comparative Zoology at Harvard University (prefix MCZ.IP), Cambridge, USA, and the Natural History Museum of Utah (prefix UMNH.IP), Salt Lake City, USA.

### 2.3. Imaging

The specimens were photographed wet under cross-polarized illumination using a Nikon D5500 DSLR fitted with a Nikon 40 mm DX Micro-Nikkor lens (general views) or a Zeiss Axiocam 208 colour camera mounted on a Zeiss Stemi 305 microscope. Interpretative drawings were created based on photographs using Photoshop CC, the software that was also used to produce all the figures, except the final version of the reconstruction of the new taxon produced by the palaeoartist Franz Anthony.

### 2.4. Compositional analysis

Large-area energy dispersive spectroscopy (EDS) was performed using a Tescan Vega GMU variable pressure scanning electron microscope equipped with two Bruker XFlash 5030 X-ray detectors on three specimens. Mapping data were acquired at a 15 mm analytical working distance and an accelerating voltage of 20 keV. All maps were acquired from the native fossil (with no mounting or surface coatings) at a chamber pressure of 20 Pa to minimize potential sample damage.

### 2.5. Terminology

The terminology used herein follows that of [27] and [51].

## 3. Results

### 3.1. Systematic palaeontology

Phylum PORIFERA Grant, 1836

Unranked SILICEA Gray, 1867

Total-group HEXACTINELLIDA

Family uncertain

Genus *Polygoniella* gen. nov.

#### 3.1.1. Diagnosis

Obconical, moderately thick-walled sponge (wall thickness up to half the flattened radius). Broad (width up to 15% of sponge diameter), prismatic exhalant canals surrounded by small cavities. Thin, dense choanosomal skeleton around exhalent canals composed of hexactins or derivatives forming an irregular, polygonal-prismatic skeletal arrangement. Internal canals are open internally and lined with a thin layer of triaxons. The outer layer of hexactins and derivatives includes long distal rays, serving as pleuralia and simple (non-anchorate) basalia. Sparse vertical marginalia surround osculum.

#### 3.1.2. Type species

*Polygoniella turrelli* gen. et sp. nov. (by monotypy).

#### 3.1.3. Etymology

From Greek *polygōnos*, meaning ‘many-angled’, in reference to the predominantly polygonal pattern of the skeleton.

#### 3.1.4. Remarks

Marjum reticulosans include the genera *Diagoniella*, *Hintzespongia*, *Protospongia*, *Testiispongia* and *Valospongia* [52,53]. Among these taxa, *Polygoniella* gen. nov. is reminiscent of small specimens of *Diagoniella* (including the probable synonym *Protospongia*) in gross morphology, to the point that we believe that some of the specimens provisionally assigned to *Diagoniella* by Rigby [52, fig. 6*c*] may belong to the new taxon. However, *Polygoniella* gen. nov. differs from *Diagoniella* in the presence of a thick cavernous wall with a polygonal choanosomal skeleton and well-defined marginalia. The latter organization strongly contrasts with the simple thin wall of *Diagoniella*, which includes only a single layer of spicules that form perfect quadrules and are devoid of prostalial or gastral rays.

*Polygoniella* gen. nov. fossils can also be mistaken for small individuals of *Valospongia*, especially as the latter taxon similarly possesses a multi-layered wall and is often preserved as carbon-rich remains in the same stratigraphic horizons of the Gray Marjum locality. The overlapping of layers of spicules may also create a pattern of subcircular structures in both taxa. However, *Polygoniella* gen. nov. lacks the distinct dermal mounds and keg-like shape characteristic of *Valospongia*. The wall cavities of *Polygoniella* gen. nov. are also noticeably more complex and recall the exhalant canals of modern hexactinellids.

*Polygoniella turrelli* sp. nov.

#### 3.1.5. Diagnosis

As for the genus.

#### 3.1.6. Etymology

In honour of the American artist James Turrell, Pomona College class of 1965, best known for his skyspaces, viewing platforms open to the sky through elliptical or rectangular openings in the ceiling.

#### 3.1.7. Material, locality and horizon

**Figure 1.**
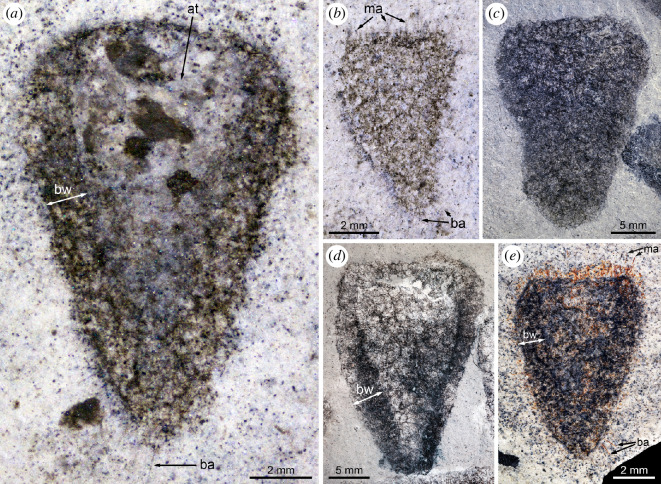
General morphology of *Polygoniella turrelli* gen. et sp. nov. from the Drumian Marjum Formation at the Gray Marjum Quarry in the House Range of Utah, USA. All images are general views of specimens immersed in dilute ethanol. (*a*) Holotype (MCZ.IP.199049) showing the basalia, a well-exposed atrial cavity, and the thick body wall. (*b*) MCZ.IP.199052 showing the external surface of the body wall, basalia and marginalia. (*c*) UMNH.IP.6425-07 showing the external surface of the body wall. (*d*) UMNH.IP.6425-09 showing the thick body wall. (*e*) MCZ.IP.199056 showing the thick body wall, and basalia and marginalia preserved as iron oxides. Abbreviations: at, atrial cavity; ba, basalia; bw, body wall; ma, marginalia.

**Figure 2.**
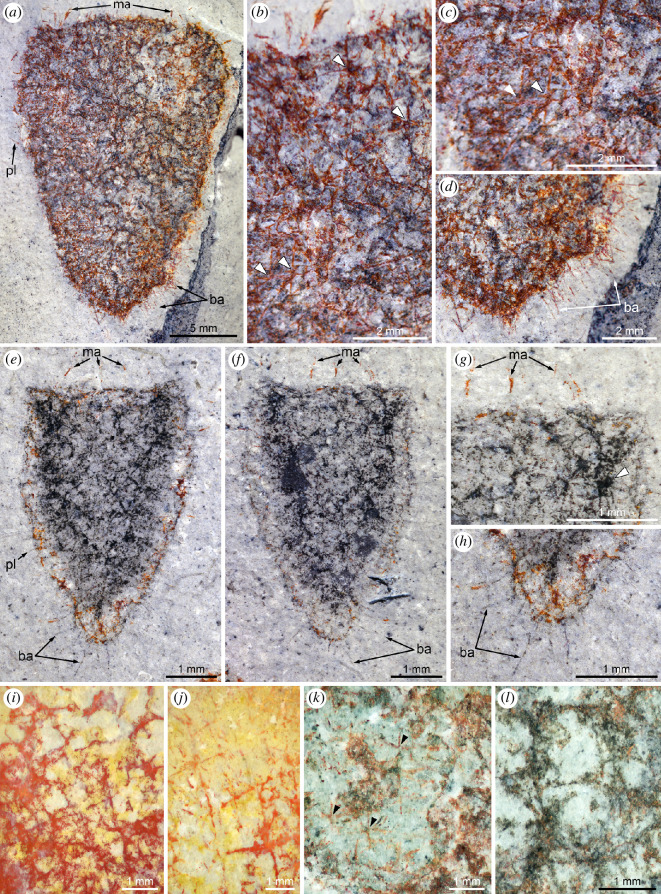
Skeleton of *Polygoniella turrelli* gen. et sp. nov. All images are of specimens immersed in dilute ethanol. (*a–d*) MCZ.IP.199410. (*a*) General view showing basalia, marginalia and pleuralia. (*b–d*) Detailed views of hexactins (arrowheads) (*b,c*) and basalia (*d*). (*e–h*) MCZ.IP.199417. (*e,f*) General views of part (*e*) and counterpart (*f*) showing the basalia, marginalia and pleuralia. (*g,h*) Detailed views of marginalia of counterpart (*g*) and basalia of part (*h*); note the choanosomal skeleton in (*g*) (arrowhead). (*i,j*) MCZ.IP.201080, detailed views showing the outermost skeletal layer of irregular to roughly diagonal arrangements of hexactins. (*k*) MCZ.IP.199627, detailed views showing hexactins (arrowheads). (*l*) MCZ.IP.199612, detailed view showing the inner skeletal layer and its fine non-fused spicules forming almost circular structures. Abbreviations: ba, basalia; ma, marginalia; pl, pleuralia.

**Figure 3.**
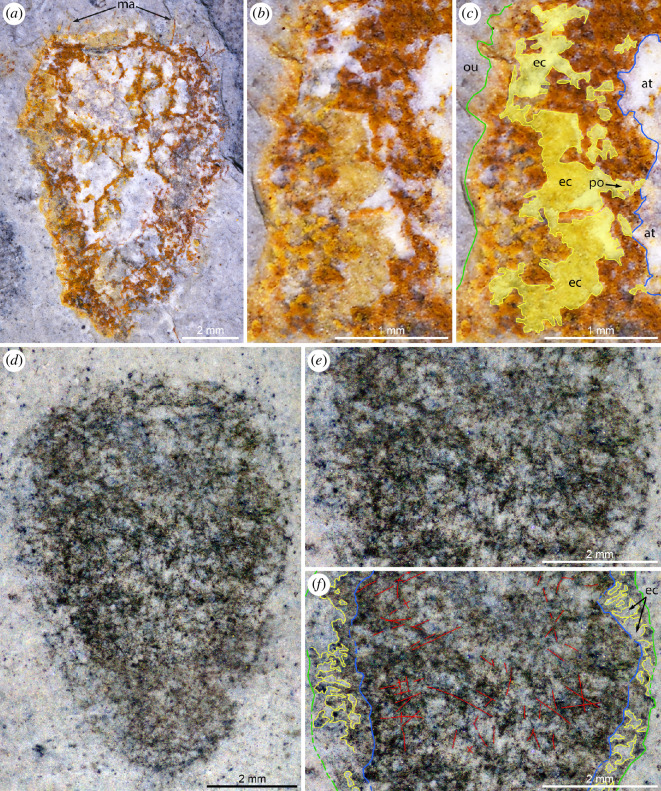
Internal structure of the body wall in *Polygoniella turrelli* gen. et sp. nov. All images are of specimens immersed in dilute ethanol. (*a–c*) MCZ.IP.199411. (*a*) General view showing marginalia. (*b*) Detailed view of internal structure of the body wall. (*c*) Annotated version of (*b*). (*d–f*) MCZ.IP.199044. (*d*) General view. (*e*) Detailed view of internal structure of the body wall. (*f*) Annotated version of (*e*). The aquiferous system (yellow), atrial wall (blue), dermal wall (green) and some spicules (red) are represented. Abbreviations: at, atrium; ec, exhalant canal; ma, marginalia; ou, outer environment; po, pore canal.

**Figure 4.**
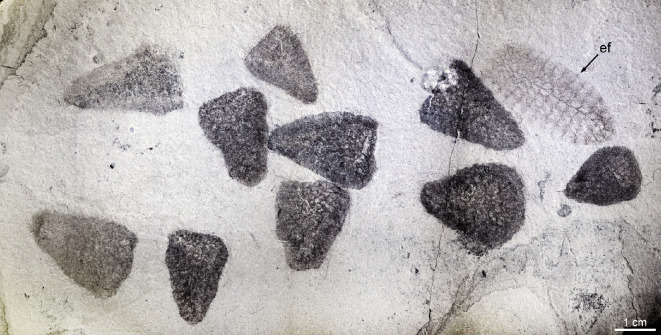
Cluster of *Polygoniella turrelli* gen. et sp. nov. Slab preserving ten individuals of the new poriferan taxon (UMNH.IP.6425-01–UMNH.IP.6425-10) and an enigmatic new fossil taxon (UMNH.IP.6425-11). Note the size and shape variations. Abbreviation: ef, enigmatic fossil taxon.

The material studied comprises *ca* 200 fossil specimens (complete and fragmentary specimens in roughly equal proportions). Of them, the holotype (MCZ.IP.199049; figure 1*a*), two paratypes (MCZ.IP.199052 and MCZ.IP.199056; figure 1*b,e*, respectively), and six additional specimens (MCZ.IP.199044, 199410, 199411, 199417, 199612, 199627, 201080; figures 2 and 3) from the type horizon at the type locality, along with ten specimens with no precise geographic information (UMNH.IP.6425-01 to 6425-10; figures 1*c,d* and 4) are illustrated (see electronic supplementary material, figures S2–S4, for illustrations of additional specimens); light-grey shale of the middle Marjum Formation, middle Cambrian (Miaolingian: Drumian), *Ptychagnostus punctuosus* agnostoid biozone; Gray Marjum locality, House Range of Utah, USA (electronic supplementary material, figure S1).

#### 3.1.8. Description

All the specimens studied exhibit similar proportions, with the height ranging from 3.5 to 28 mm (mean: 9 mm) and the width from 2 to 21 mm (mean: 6 mm). 25% of individuals are less than 7 mm in body height, 47% of them measure between 7 and 10.5 mm, and 28% of them exceed 10.5 mm in body height. Individuals are typically found in pairs or clusters (figure 4) rather than isolated.

Skeleton primarily composed of hexactins or derivatives, some with elongate distal rays forming pleuralia (1−2 mm), basalia (1−2 mm) and marginalia (1 mm) (figure 2; electronic supplementary material, figure S4). The oscular margin is three times wider than the base, and the maximum width is usually slightly below it. Marginalia is sparse, separated and composed of vertical distal rays of triaxons with otherwise reduced rays (figure 2*a*,*b*,*e*–*g*; electronic supplementary material, figure S4*a*,*b*).

The outermost skeletal layer—likely the hypodermal rather than dermal skeleton—is most recognizable when superimposed on the centre of the exhalant canals and near the upper margin of the sponge (figure 2*b*,*c*; electronic supplementary material, figure S2). This layer consists of irregular to roughly diagonal arrangements of hexactins or derivatives, with angles less than 40° relative to sponge vertical axis (figure 2*i*,*j*). The ray length is up to 1.9 mm. In some specimens, larger spicules identified as basalia diverge from the base downward at acute angles (figure 2*a*,*d*–*f*,*h*; electronic supplementary material, figure S4*e*–*h*). These longer rays are projecting distal rays of hexactins embedded into the outer layers of the basal part of the sponge. Pleuralia extends from the lateral margins along the entire height of the sponge (figure 2*a*,*e*; electronic supplementary material, figure S4*c*,*d*); they are similar to the basalia, except that they are slightly inclined towards the top of the sponge.

The choanosomal body wall is up to 2 mm thick. Its non-fused spicules form an irregular polygonal pattern around large cavities interpreted as exhalant canals (figures 1, 2 and 3; electronic supplementary material, figure S3). Fine spicule rays border the margins of the canals, forming an almost circular structure when superimposed, appearing as though they were diactins. The presence of diactins or monactins, however, has not been confirmed, even when using low-angle illumination and high magnification.

The holotype MCZ.IP.119049 is 13.5 mm tall (figure 1*a*). Its width increases from 7.5 mm (at the oscular margin) to 8 mm (2.5 mm below the oscular margin), and then progressively decreases down to 2 mm at the base. The pleuralia and basalia project up to 1 mm beyond the body wall. The latter wall includes two layers of hexactins or derivatives, some with rays up to 0.5 mm. The paratype MCZ.IP.119052 measures 7.5 mm in height and 5 mm in maximum width (figure 1*b*). It displays regularly spaced (25 µm) prominent marginalia and basalia, with rays up to 0.8 mm. At the oscular margin, a dense arrangement of pseudo-quadrules of hypodermalia is present, due to superimposition of the outer layer of the wall and the internal canals. The preservation of this net-like skeletal organization is common to most specimens. The paratype MCZ.IP.119056 measures 8 mm in height and 5.5 mm in maximum width (figure 1*e*). Its 1-mm-thick wall includes regularly spaced (0.025 mm) prominent marginalia and basalia, with rays up to 0.8 mm. The dense arrangement of pseudo-quadrules of hypodermalia at the oscular margin is also preserved in this specimen.

Two specimens from the collections of the Natural History Museum of Utah (precise geographic origin unknown) are also illustrated herein. UMNH.IP.6425-09 is one of the largest known individuals, reaching 26.5 mm in height and 18.5 mm in width (figure 1*d*). Despite a dense carbonaceous cover, the prismatic-polygonal spicule arrangement around the exhalant canals is visible in the choanosomal layer, with spicule rays up to 0.68 mm long. Weakly preserved basalia and prostalia are observed. The individual has preserved some three-dimensionality in a region bordering the oscular margin, the two sides of the obliquely compressed sponge being topographically separated by a sediment infill. Lastly, UMNH.IP.6425-07 measures 24 mm in height and 17 mm in maximum width and displays an obliquely compressed osculum (figure 1*c*).

**Figure 5.**
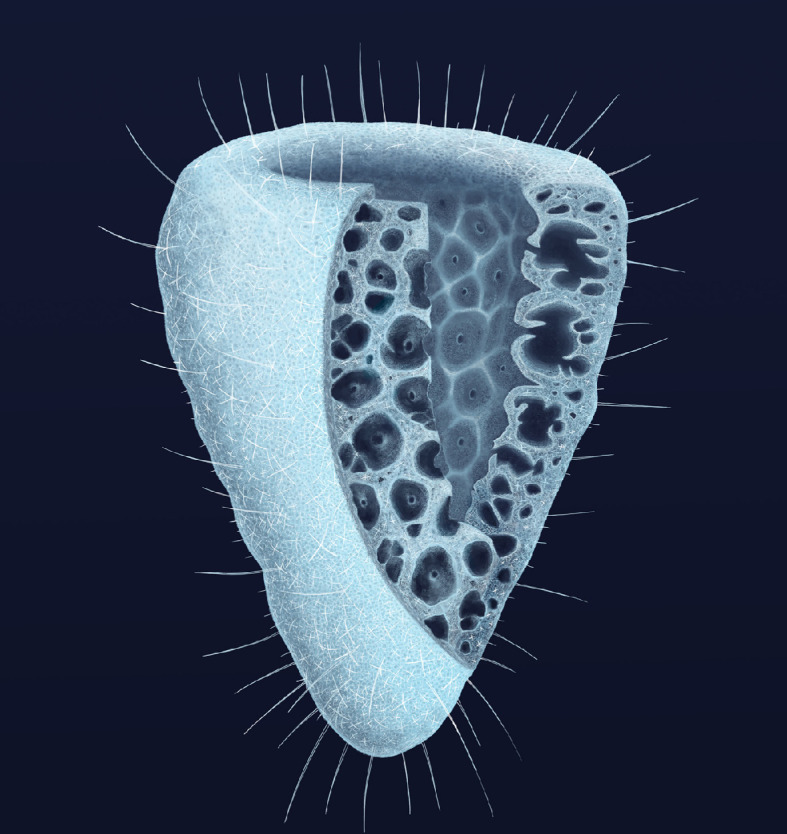
Artistic reconstruction of *Polygoniella turrelli* gen. et sp. nov. from the Marjum Biota. Artwork by Franz Anthony, modified from a drawing by J.B. Copyright Harvard University.

A morphological reconstruction of the new taxon is presented in figure 5.

#### 3.1.9. Remarks

The change in orientation differentiating pleuralia from basalia is observed in multiple specimens and may suggest that the bottom one-quarter to one-third of the body was attached to the seafloor or even embedded within it.

#### 3.1.10. Preservation

**Figure 6.**
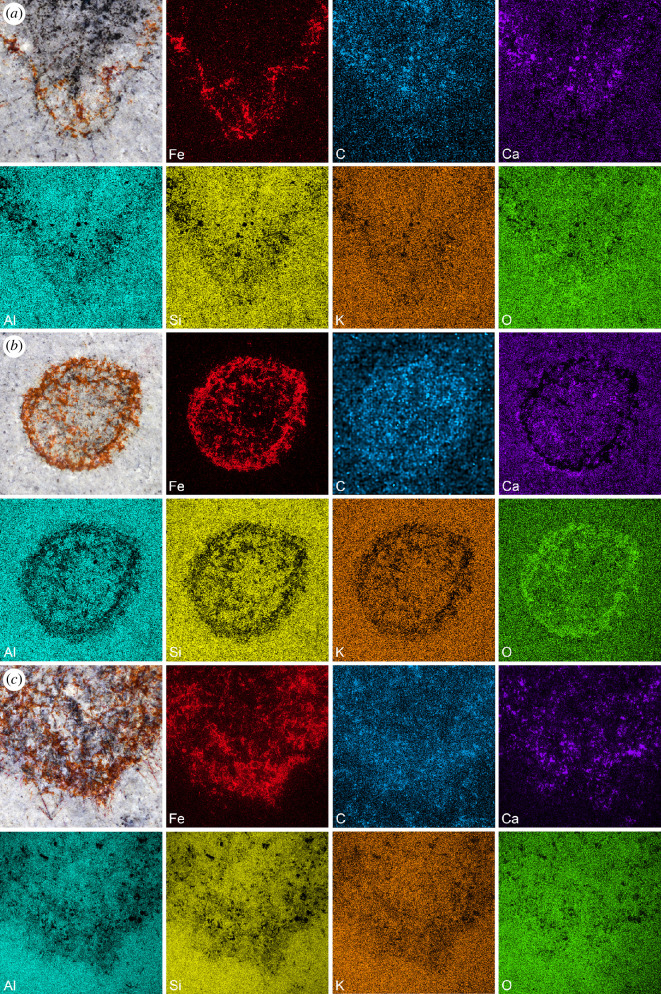
Elemental composition of specimens of *Polygoniella turrelli* gen. et sp. nov. from the Marjum Biota. A detailed view of the analysed area (upper left panel) and the elemental maps of the main elements detected by EDS in the fossil (subsequent upper panels) and the surrounding matrix (lower panels) are presented for each specimen. (*a*) MCZ.IP.199417, specimen composed of two spatially separated materials, a dark grey material (carbonaceous remains) in the inner region and a red material (spicules replaced by iron oxides) in the outer region. (*b*) MCZ.IP.199419, specimen showing a broad overlap of the two materials. (*c*) MCZ.IP.199410, specimen showing a broad overlap of the two materials. Note that the composition of matrix is suggestive of clay mineral(s) with some amount of calcium carbonate.

EDS analysis of the three specimens confirms that the mudstones comprising the Gray Marjum strata are rich in clay minerals (Al, K, Mg, Na, O and Si) and, to a lesser extent, calcium carbonate (C, Ca and O), a composition typical of Burgess Shale-type deposits [40] (figure 6*a*–*c*; electronic supplementary material, figure S5). Two types of preservation are observed among the specimens (figures 1—4; electronic supplementary material, figures S2–S4). In most individuals, a dark grey material obscures the construction of the underlying spicule layers, but allows the identification of soft structures (e.g. exhalant canals), particularly at the margins (figure 3*d–f*). This dark grey material is noticeably richer in C, and occasionally Ca, compared to the matrix according to EDS (figure 6*a*; electronic supplementary material, figure S5). Some specimens are partly or entirely weathered, with red stains highlighting the organization of the biomineralized skeleton, particularly that of the choanosomal layer (figure 3*a–c*). EDS shows that this red material replacing the spicules (figure 6*b,c*; electronic supplementary material, figure S5), or a part of them (figure 6*a*), is considerably enriched in Fe relative to the matrix and to the parts of the fossils that are preserved as a dark grey material. We infer that the Fe-rich phase is likely an iron oxide derived from weathering of sedimentary pyrite in the bulk rock, rather than of pyrite originally associated with the fossils. No pyrite is present in the unweathered cores of the fossils. One of the three analysed fossils exhibits small patches of barite scattered over the whole surface of the specimen as inferred from enrichments of Ba, S and O observed by EDS (electronic supplementary material, figure S5). This unique occurrence of barite most likely results from diagenesis or surface alteration, although accumulation of Ba during the life of this sponge [54] or during early diagenesis of organic matter cannot be ruled out at this stage. Regardless of weathering state, microscopic characteristics of the spicules (e.g. internal structure, ray terminations) could not be observed in any of the specimens, as they were lost to weathering or obscured by the carbonaceous film.

## 4. Discussion

### 4.1. Morphological disparity in early poriferans

Early Palaeozoic exceptionally preserved biotas include a great variety of sponges (e.g. [43,55–59]), many of which cannot be readily accommodated in the classification scheme developed from the study of modern representatives. Despite recent progress [27,60,61], the path to a universal systematic scheme for the whole phylum, one that would fully acknowledge the great disparity exhibited by its early representatives and discourage the current overemphasis put on some characters (e.g. spicule geometry) is still paved with major difficulties. Among these challenges are the questions of spicule homology and how much of the original spicule mineralogy, microscopic features (e.g. microscleres), and soft anatomy (e.g. aquiferous system) can be preserved in fossil sponges. Individual species with exceptional preservation of one or more of these aspects may radically change our understanding of the group as a whole.

Most groups of fossil and living spiculate sponges are fundamentally different from the new genus (see [27] for a detailed summary of the characters of extant sponge groups, and their interpreted stem groups). During the early Palaeozoic, offshore benthic marine environments were dominated by two groups of thin-walled sponges, the ascosponges and the reticulosans. In both groups, the biomineralized skeleton is usually single-layered and may include hexactins, the character putatively diagnostic of the class Hexactinellida.

The ascosponges—an early diverging and exclusively fossil clade of sponges, which are typically the most abundant forms in Burgess Shale-type faunas—have a thin body wall with a dominant longitudinal spicule arrangement of monaxons, sometimes with a secondary transverse component [29]. Even in some early forms where subsidiary, short-rayed hexactins have been reported [29,30], the skeletal architecture is entirely distinct from that of *Polygoniella* gen. nov. Complex body walls are also present in spiculate demosponges, but in that case the skeleton is dominated by either monaxons or tetractins, usually very small. Triaxons appear to have been present in their earliest stem group [27], but these sponges are already significantly different in the dominant spicules and the body wall architecture. Given that the original mineralogy of spicules in *Polygoniella* cannot be determined, a relationship to the Calcarea should also be considered. No triaxons are present in extant Calcarea, but they were present in the presumed stem-group example (or basal stem-silicean) *Eiffelia* [34], and calcitic hexactins were present in the enigmatic, somewhat hexactinellid-like *Carduispongia* [28]. *Eiffelia*, however, differs fundamentally in its dominant hexaradiate spicules in a thin body wall, whereas *Carduispongia* has a well-preserved three-dimensional architecture that is very different from that seen in *Polygoniella*, and more similar to the sylleibid architecture of some extant calcareans and homoscleromorphs. Given these fundamental differences in skeleton and body-wall architecture, the only groups that require a close comparison are reticulosans and hexactinellids.

Cambrian reticulosans possess stauractins and/or hexactins typically forming (semi-)reticulate or quadruled arrangements (orthogonal or diagonal). This has led some authors to consider all reticulosans as hexactinellids (e.g. [62–64]), but their overall simple anatomies provide no supporting evidence for that interpretation. Most reticulosans have a thin single-layered wall with a variably well-expressed quadruled organization of spicules (e.g. *Diagoniella*, *Testiispongia* and *Valospongia*), and even when they add an internal skeletal layer to a *Diagoniella*-like external one as in *Hintzespongia* [65], the two layers are apparently unconnected by skeletal material. Critically, there is no clear evidence of internal cavities within the body wall of these early sponges and an asconoid construction, where water filtration occurs within the choanoderm-lined atrium, appears likely for at least the Protospongiidae [62]. Only a handful of Ordovician reticulosans have hitherto been documented to possess an anatomical complexity suggestive of closer relationships to extant hexactinellids (e.g. [56]) namely the Early Ordovician–Silurian *Cyathophycus* [66] and the Middle Ordovician–Carboniferous *Teganium* [67]. Both taxa have been reconstructed as possessing a two-layered wall with small simple cavities. The structure of *Polygoniella* from the Marjum Formation is similar to these examples in fundamental morphology (multiple spicule layers with internal cavities), but more complex in the three-dimensional body wall architecture.

### 4.2. Hexactinellid affinities of *Polygoniella* gen. nov

Close examination of the architecture of *Polygoniella* gen. nov. reveals a striking difference relative to other Cambrian sponges. Its hypodermal skeleton is exclusively composed of hexactine derivatives (unlike the monaxon-dominated skeleton of ascosponges), but these spicules do not form the regular reticulum of quadrules characterizing protospongiids and early dictyospongioids [27,30]. Instead, the hypodermal skeleton appears disordered in the new taxon (figure 3*b*,*c* and 4*f*), with only traces of a quadruled arrangement near the oscular margin. Such disordering is also present in some other Cambrian reticulosans [52,53], but in these examples a thin and generally simple body wall is retained. The presence of an internal (choanosomal) skeletal layer and the thick-walled morphology resulting from it (figures 1*a*,*d*,*e*, 3*e*,*f* and 4; electronic supplementary material, figure S3) are also rare in Cambrian sponges, but it is the complexity of this layer, with its large cavities (exhalant canals; figure 3) surrounded by internal walls (figure 2*g*), that makes it so different from other Cambrian sponges. Instead, the architecture is only comparable with that of modern hexactinellids.

Because of the abundance of specimens and the range of preservation they display, the three-dimensional organization of the aquiferous system of *Polygoniella* can be reconstructed with reasonable confidence (figure 5). This arrangement is most reminiscent of a syconoid construction, where water that enters through minute dermal ostia is filtered within the body wall in choanoderm-lined/flagellated chambers surrounding large exhalant canals, and eventually leaves through a large osculum after passing through the atrium (figure 5). The presence of flagellae lining the filtering chambers cannot be ascertained in these carbonaceous compression fossils, but the other elements characterizing this architecture of the body wall are well established (figures 1 and 3; electronic supplementary material, figure S4). Hexactinellids in general, including numerous extant representatives of the subclasses Amphidiscophora and Hexasterophora [26], possess a moderately complex aquiferous system that does not strictly fall within the ascon-sycon-leucon classification of other sponges [68], but in many taxa is closest to a leuconoid organization. Numerous variations documented in various modern taxa more closely resemble the organization observed in *Polygoniella* (e.g. choanoderm-lined exhalant chambers in *Euplectella* in Schulze [69]; and *Farrea* in Reiswig & Mehl [70]), and this body wall architecture seems to represent a fundamental trait of the class Hexactinellida. It is particularly well illustrated by [71] in taxa such as *Chonelasma*, *Hyalonema* and *Polylophus*: large bell-shaped exhalant canals are separated from the atrial cavity by a gastral wall that sometimes exhibits large perforations, and these canals are flanked by flagellated chambers that are typically *ca* 100 μm in diameter.

The fact that this architecture is known in representatives of the two main clades of extant hexactinellids suggests that it was inherited from their last common ancestor. In the absence of a phylogenetic framework, this character alone does not permit a definitive assignment of *Polygoniella* to crown-group hexactinellids; this would require the description of additional, presumably more derived features (e.g. microscleres, skeletal fusion, diagnostic spicular morphologies) indicative of close relationships with one or the other subclass of modern hexactinellids. Due to their minute sizes, it is possible that microscleres were present in this Cambrian taxon, but not preserved or observed, and demonstrating skeletal fusion may also be difficult in two-dimensionally preserved and demineralized fossils. However, the absence of pinular spicules (or other obvious dermalia) may suggest a placement of *Polygoniella* close to, but stem-ward of crown-group hexactinellids (table 1).

**Table 1.** Oldest records of some characters expected to be present in the last common ancestor of crown-group Hexactinellida. S/C indicates whether the character is thought to have been present in the stem group (S) or to have originated in the crown group (C) of Hexactinellida, based on whether they are present in most living representatives.

character	S/C	taxon	age	remark	reference
hexactin	S	indeterminate	Cambrian 1 (Fortunian)	also present in non-hexactinellid taxa	[72]
multi-layered wall	S	*Hintzespongia*, *Polygoniella*	Cambrian 6 (Drumian)	also in non-hexactinellid stem-group taxa; polyphyletic	[65], herein
intra-wall cavities	S	*Polygoniella*, *Valospongia*	Cambrian 6 (Drumian)	—	[52], herein
hypodermal pentactin layer	S	*Heminectere*?	Ordovician 5 (Sandbian)	widespread in early reticulosans, but hypodermal position not demonstrated	[56]
hexactinellid-type microscleres	S?	indeterminate	Ordovician 1 (Tremadocian)	isolated microscleres	[32]
pinulate spicules	C?	indeterminate	Ordovician 1 (Tremadocian)	isolated megascleres	[32]
skeletal fusion	C	*Casearia*	Devonian 3 (Emsian)	—	[73]

Whatever its precise position within the hexactinellid lineage, *Polygoniella* unambiguously documents what the moderately complex construction of the Middle Ordovician *Teganium* had suggested so far: a complex aquiferous system had evolved early in hexactinellid history. *Polygoniella* demonstrates that the acquisition of a syconoid-like (or *Farrea*-like) construction in hexactinellids predates by at least 13.6 Myr the oldest record of hexactinellid-specific microscleres (Lower Ordovician) [32] and by 61.9 Myr the oldest known articulated crown-group hexactinellid (Ordovician) [33], according to the stratigraphic chart of [71].

## 5. Conclusion

Poriferan classification has traditionally relied on the observation of sets of characters that distinguish extant clades, but this systematic framework has proved to be largely inapplicable to early members of the group. The body organization of Cambrian sponges is rudimentary compared to their modern counterparts, and yet many of them possess hexactins, a supposedly diagnostic feature of the class Hexactinellida. *Polygoniella turrelli* gen. et sp. nov. displays a uniquely complex body wall organization amongst Cambrian sponges, with a thin hypodermal layer containing an irregularly arranged hexactin-based skeleton, and a thick choanosomal layer comprising large exhalant canals surrounded by small (flagellated?) chambers. This construction is strongly reminiscent of that of various modern amphidiscophoran and hexasterophoran hexactinellids, but in the absence of a clear phylogenetic framework and additional derived features (e.g. microscleres), it remains unclear whether *Polygoniella* belongs to the crown-group of Hexactinellida or its uppermost stem-group.

The discovery of *Polygoniella* further illustrates the great diversity of body organization within Cambrian sponges. The Marjum poriferan fauna includes representatives with a mono-layered body wall and a monaxon-dominated skeleton (e.g. leptomitiids), a mono-layered body wall and a well-organized hexactin-dominated skeleton (e.g. *Diagoniella*), a thin bi-layered body wall (e.g. *Hintzespongia*), and a thick multi-layered body wall with a complex alveolar organization (e.g. *Polygoniella*) [47,48]. This mixing of anatomically simple and complex forms within the same biota is rather typical of Cambrian animal communities. Somewhat echoing the description of co-occurring stem-group and crown-group members in other taxa (e.g. arthropods, cnidarians and annelids) [73–76], it attests to the rapidly changing evolutionary landscape that characterizes the Cambrian period. How this important anatomical disparity of early sponges impacted the ecological structures of poriferan communities remains to be investigated, but the Marjum Biota and its 25 poriferan species suggest that it had no adverse effect on taxonomic diversity.

### 5.1. Data and software availability

Nomenclatural acts relating to the new taxon are registered on ZooBank:

urn:lsid:zoobank.org:pub:13AC2E6F-F064-43CC-A600-2AEFF5064D90 (publication),

urn:lsid:zoobank.org:act:9EBDB63A-ABB2-4AC3-B7C7-4560C2E52064 (genus),

urn:lsid:zoobank.org:act:B6DC5F91-1ADD-4683-8FD3-7BABC22941A4 (species).

## Data Availability

Fossil specimens are stored in the Harvard Museum of Comparative Zoology (MCZ.IP), Cambridge, USA, and the Natural History Museum of Utah (UMNH.IP), Salt Lake City, USA. The list of studied specimens and five additional figures are available as supplementary material [77].
